# Two Pediatric Cases of Food Protein-Induced Enterocolitis Syndrome to Soy Eaten as an Infant Food

**DOI:** 10.7759/cureus.38556

**Published:** 2023-05-04

**Authors:** Keisuke Maeda, Yoshiki Kusama, Yukari Atsumi, Tadamori Takahara, Katsunori Kamimura

**Affiliations:** 1 Department of Pediatrics, Hyogo Prefectural Amagasaki General Medical Center, Hyogo, JPN; 2 Department of Pediatric Allergy, Hyogo Prefectural Amagasaki General Medical Center, Hyogo, JPN

**Keywords:** shock, fermentation, infant food, soy, tofu, food protein-induced enterocolitis syndrome

## Abstract

Food protein-induced enterocolitis syndrome (FPIES) is a non-IgE-mediated food allergy that can be caused not only by infant formula but also by infant food. Herein, we report two pediatric cases of FPIES to solid soy foods, such as tofu. The patients presented with repetitive vomiting after eating the trigger food as infant food. Although both cases promptly recovered following the cessation of the trigger food, one case required rapid intravenous hydration for compensated shock. Both cases were diagnosed with FPIES to soy based on the typical presentation and parental interviews regarding food exposure. One case had a positive response to an oral food challenge for tofu, and both cases were negative for soy-specific IgE. One of our cases did not develop FPIES from fermented soy products despite having soy-triggered FPIES. The fermentation process may reduce the allergenicity of soy, but further evidence is required to confirm this hypothesis. There are various trigger foods for solid food FPIES (SFF), and these differ among countries. Solid food FPIES to soy is more common in Japan than in other countries due to the frequent use of tofu in infant food. Increased international awareness of the possibility of tofu-triggered FPIES may be warranted due to the rising global use of tofu in infant food.

## Introduction

Food protein-induced enterocolitis syndrome (FPIES) is a non-IgE-mediated food allergy that primarily affects infants and children, and typically presents as repetitive vomiting without dermal or respiratory symptoms [1]. While the pathophysiology of FPIES is not fully understood, it is believed to be the result of gut inflammation in response to an antigenic food protein, which then causes increased intestinal permeability [2]. FPIES can become severe and lead to shock, which requires differentiation from sepsis during the diagnostic process. Despite its potential severity, high-quality studies on FPIES are scarce, and the paucity of evidence is regarded as a problem [2]. In the past, most cases of FPIES were thought to be caused by cow’s milk or soy formula ingestion (cow’s milk and soy FPIES (CSF)) in early infancy and sometimes induced by breast milk containing those proteins. However, infant foods have come to be regarded as an important trigger of FPIES following the publication of a case series of solid food FPIES (SFF) in 2003 [3]. Rice and oats are recognized as major causes of SFF [4], but other foods can also trigger this condition [5]. Cases of FPIES to hen’s eggs, especially egg yolk, have recently increased [6]. Although soy is a major trigger food for CSF [2], few cases of soy-triggered SFF have been described. Herein, we report two pediatric cases of SFF to traditional Japanese soy foods, such as tofu and kinako.

## Case presentation

Case 1

**Figure 1:**
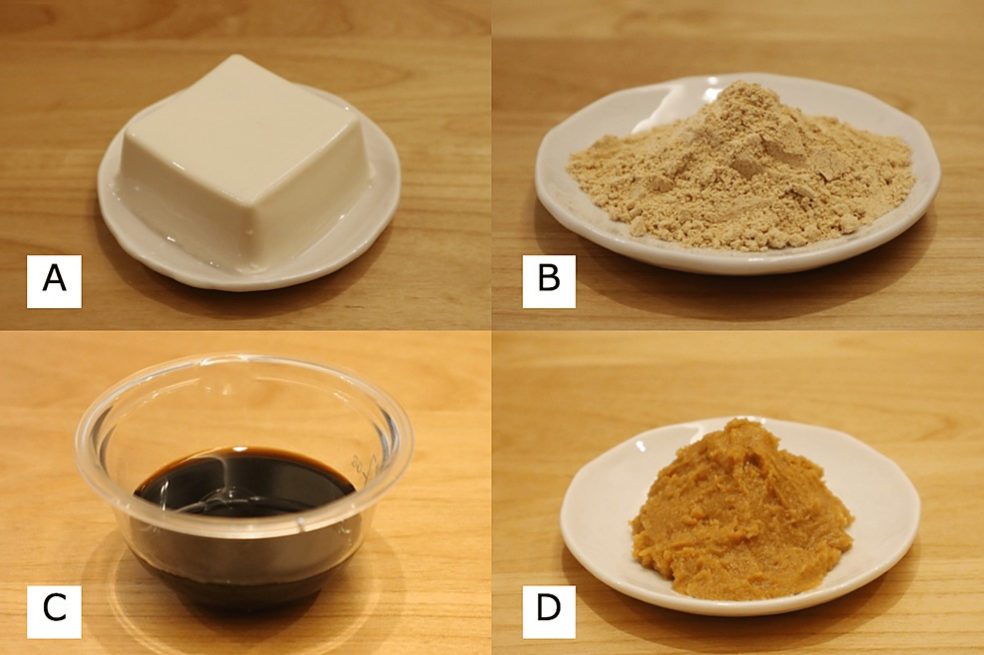
Photos of traditional Japanese foods made from soy A: tofu, B: kinako, C: shoyu, D: miso All photos were taken by Keisuke Maeda.

**Figure 2:**
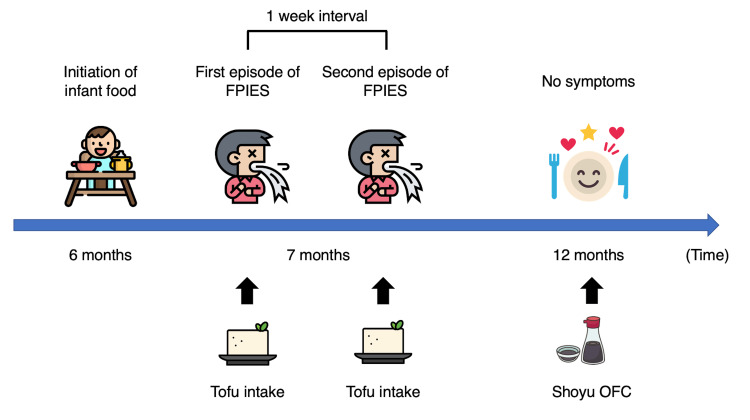
Clinical course of case 1 FPIES: food protein-induced enterocolitis syndrome, OFC: oral food challenge This figure was originally created by Yoshiki Kusama. All icons were license-free and were designed by Freepik and distributed by Flaticon (https://www.flaticon.com).

The first case was a seven-month-old male who was raised on breast milk and cow’s milk formula. His parents introduced solid foods at six months of age. The patient had previously eaten approximately 15 g of tofu (Figure 1) once and had no history of consuming other soy products before this presentation; he had shown no prior indication of food allergy. He presented to the emergency department of our hospital with repetitive vomiting and pallor 1.5 hours after consuming infant food. His vital signs were as follows: axillary temperature of 36.8°C, respiratory rate of 24 breaths/minute, heart rate of 125 beats/minute, and oxygen saturation of 98% (blood pressure could not be measured due to patient refusal). Physical examination found no abnormalities, and laboratory tests did not detect any soy-specific IgE (Table 1). At the time of consultation, five hours had passed since food intake, and the patient’s vomiting had resolved spontaneously. Therefore, he was sent home without hospitalization despite the unknown etiology of his symptoms. For one week after the visit, the patient did not develop any similar symptoms. However, he experienced another episode of repetitive vomiting and pallor a week later. Through interviews with the patient’s parents, the examining physician deduced that both episodes were caused by tofu intake (approximately 15 g of tofu in both episodes), as the patient did not consume tofu during the non-symptomatic period. The patient was diagnosed with FPIES to soy, and his parents were instructed to eliminate soy from his diet. Thereafter, he experienced no further symptoms. Five months after the second FPIES episode, the patient underwent an oral food challenge for soy sauce (shoyu) (Figure 1). As he did not show a positive response to the test, he was cleared for shoyu consumption. We plan to perform an oral food challenge for tofu itself a year after the second FPIES episode. The clinical course of this case is presented in Figure 2.

**Table 1:** Results of laboratory testing in cases 1 and 2 ALST: allergen-specific lymphocyte stimulation test

Test	Case 1	Case 2
Leukocyte count (10^9^/μL)	10.4	11.1
Neutrophil count (10^9^/μL)	2.8	2.2
Eosinophil count (10^9^/μL)	0.5	0.2
Percentage of neutrophils (%)	27	20
Percentage of eosinophils (%)	4.5	2
C-reactive protein (mg/dL)	0.87	0.02
Total IgE (IU/mL)	1	10
Soy-specific IgE (UA/mL)	<0.10	<0.10
Gly m4-specific IgE (UA/mL)	<0.10	<0.10
ALST for lactoferrin	Not tested	Positive

Case 2

The second case involved an eight-month-old female who was breastfed exclusively. The patient’s parents introduced solid foods at six months of age, and she had no history of consuming any soy products before this presentation; she had shown no prior indication of food allergy. She presented to the emergency department of our hospital with the chief complaint of repetitive vomiting and extreme lethargy two hours after eating yogurt with roasted soy powder (kinako) (Figure 1). Her vital signs were as follows: axillary temperature of 37.2°C, respiratory rate of 30 breaths/minute, heart rate of 120 beats/minute, and oxygen saturation of 98% (blood pressure could not be measured due to patient refusal). The patient also had impaired consciousness (E2V2M5 on the Glasgow Coma Scale) and presented with peripheral coldness and livedo reticularis of the extremities. Laboratory tests did not detect any soy- or milk-specific IgE, but an allergen-specific lymphocyte stimulation test for lactoferrin yielded a positive result (Table 1).

**Figure 3:**
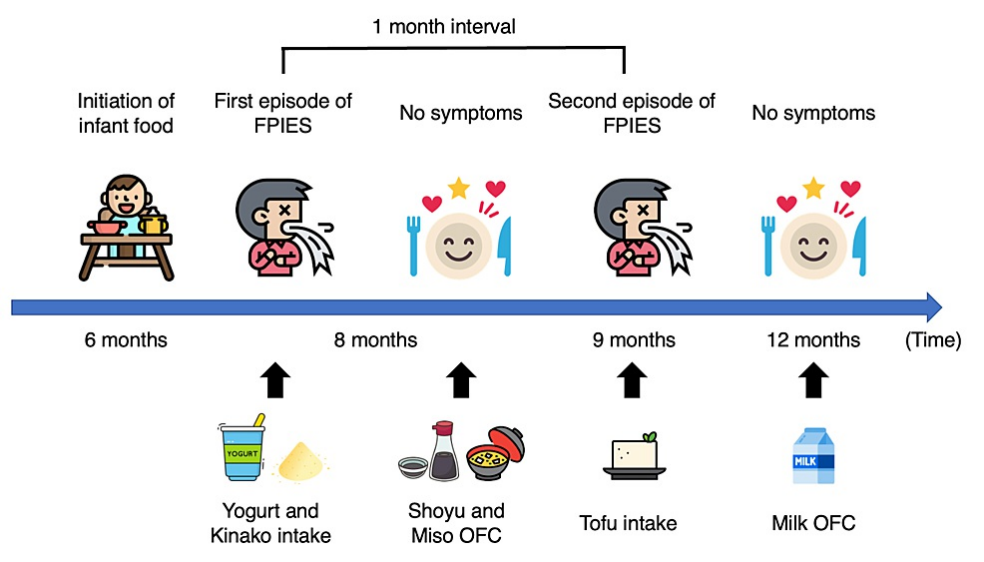
Clinical course of case 2 FPIES: food protein-induced enterocolitis syndrome, OFC: oral food challenge This figure was originally created by Yoshiki Kusama. All icons were license-free and were designed by Freepik and distributed by Flaticon (https://www.flaticon.com).

During the patient’s hospital visit, she continued to experience repetitive vomiting, lethargy, and impaired consciousness. We administered rapid intravenous hydration in response to compensated shock. Her vomiting ceased, and her condition improved. The patient was hospitalized overnight for observation and was discharged the next day after we confirmed that she had recovered and had an adequate volume of oral intake. We suspected FPIES and informed her parents to eliminate milk and soy from her diet since both yogurt and kinako are potential triggers. After the removal of these foods, the patient did not experience further symptoms. We initially considered the trigger food to be cow’s milk due to the positive result in the allergen-specific lymphocyte stimulation test for lactoferrin but also administered oral food challenges for shoyu and miso (Figure 1). Although the patient did not develop symptoms to these oral food challenges, she experienced another episode of repetitive vomiting, lethargy, and impaired consciousness after eating tofu a month later. Her parents were instructed to eliminate tofu from her diet. Thereafter, she did not have any recurrence of the FPIES symptoms. The patient showed a negative result for a cow’s milk oral food challenge performed after her one-year birthday. Then, she underwent an oral food challenge for tofu one year after the last FPIES episode and experienced repetitive vomiting and a rapid increase in serum thymus and activation-regulated chemokines. Thus, tofu continued to be eliminated from her diet. We plan to perform the second tofu oral food challenge a year after the first failed challenge. The clinical course of this case is presented in Figure 3.

## Discussion

FPIES typically occurs at the first exposure to a formula in early infancy, but there has been a recent increase in patients with SFF in Japan [7]. In contrast to IgE-mediated allergies, the diagnosis of FPIES lacks adequate laboratory-based tests, and oral food challenges should be supported by careful interviews to determine food exposure [5]. As FPIES must first be suspected before it can be diagnosed, pediatricians should be aware that SFF can occur upon the introduction of solid foods and not only during early infancy. Pediatricians should also consider the possibility of FPIES for patients presenting with repetitive vomiting after consuming infant food. Table 2 shows the diagnostic criteria for acute FPIES developed by the American Academy of Allergy, Asthma, and Immunology [2]. A diagnosis of FPIES is made when a patient meets the major criterion and at least three minor criteria. Case 1 met the major criterion and five minor criteria, and Case 2 met the major criterion and six minor criteria.

**Table 2:** Diagnostic criteria of food protein-induced enterocolitis syndrome

Major criterion
Vomiting in the 1-4 hour period after ingestion of the suspect food and the absence of classic IgE-mediated allergic skin or respiratory symptoms
Minor criteria
A second (or more) episode of repetitive vomiting after eating the same suspect food
Repetitive vomiting episode 1-4 hours after eating a different food
Extreme lethargy with any suspected reaction
Marked pallor with any suspected reaction
Need for an emergency department visit with any suspected reaction
Need for intravenous fluid support with any suspected reaction
Diarrhea in 24 hours (usually 5-10 hours)
Hypotension
Hypothermia

Although most cases of CSF are triggered by infant formula (either cow’s milk or soy), a wide variety of foods can trigger SFF, and these trigger foods differ among countries [8]. For example, rice is the most frequent cause of SFF in the USA [9], but it is rare in Italy [10]. Conversely, fish is a rare cause of SFF in the USA, but it is a common trigger food in Italy [10]. The reasons for these differences are thought to include variations in lifestyle, environment, and genetic diversity [11]. A study showed that the most common trigger food of SFF in Japan is a hen’s egg, followed by wheat and soy [7]. To our knowledge, there have been no studies that determined if soy-triggered FPIES occurs more frequently as CSF or SFF. However, a Japanese study examined 32 cases of FPIES in early infancy and reported that all were caused by cow’s milk [12]. This suggests that soy is a rare cause of CSF and that most cases of soy-triggered FPIES occur as SFF in Japan. CSF to soy is more common in the USA [13], which may stem from the frequent use of soy formula [14]. Similarly, the relatively high prevalence of SFF to soy in Japan may be due to the frequent use of tofu in infant foods. Currently, there are many websites in numerous languages recommending the consumption of tofu as an infant food based on its healthy image. There may be a need for greater international awareness that tofu is a possible trigger for SFF in infants and young children.

**Figure 4:**
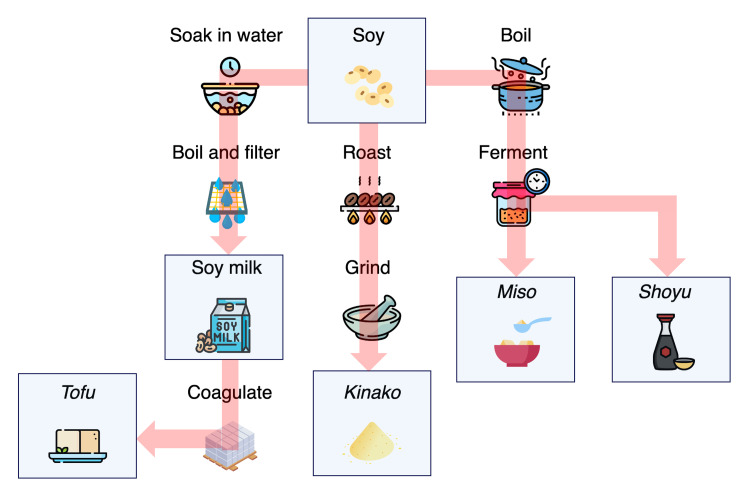
Flow of food processing for soy products This figure was originally created by Yoshiki Kusama. All icons were license-free and were designed by Freepik and distributed by Flaticon (https://www.flaticon.com).

In case 2, kinako and tofu triggered SFF, but shoyu and miso did not induce any symptoms. An important difference is that shoyu and miso, unlike kinako and tofu, are fermented (Figure 4). A previous study showed that fermentation can dramatically reduce Gly m5 and Gly m6 levels, which are major allergens associated with severe IgE-mediated soy allergy [15]. Another study reported that the IgE affinity of soy protein is reduced by 65%-99% through fermentation [16]. It is possible that the fermentation process can also reduce the allergenicity of soy in FPIES, but more studies are needed to explore this relationship and its underlying mechanisms. Because shoyu and miso are integral components of the Japanese diet, it would be highly beneficial for Japanese children if studies can confirm that the fermentation of soy products prevents FPIES.

## Conclusions

Physicians should consider SFF in the differential diagnosis of young children presenting with repetitive vomiting after being introduced to solid foods. Detailed interviews are essential when FPIES is suspected. As there is a current lack of laboratory diagnostic methods for FPIES, oral food challenges remain the gold standard for diagnosis. Trigger foods differ among countries, with tofu being a common cause of SFF in Japan. Increased international awareness of the possibility of tofu-triggered FPIES may be warranted due to the rising global use of tofu in infant food.
